# A cross-cultural interpersonal model of adolescent depression: A qualitative study in rural Nepal

**DOI:** 10.1016/j.socscimed.2020.113623

**Published:** 2021-02

**Authors:** Kelly Rose-Clarke, Eliz Hassan, Prakash BK, Jananee Magar, Delan Devakumar, Nagendra P. Luitel, Helen Verdeli, Brandon A. Kohrt

**Affiliations:** a Department of Global Health and Social Medicine, King's College London, UK; b Transcultural Psychosocial Organization Nepal, Kathmandu, Nepal; c Institute for Global Health, University College London, UK; d Global Mental Health Lab, Teachers College, Columbia University, New York, NY, USA; e Department of Psychiatry and Behavioral Sciences, And Department of Global Health, George Washington University, Washington, DC, USA

**Keywords:** Nepal, Depression, Adolescents, Interpersonal model

## Abstract

Most cross-cultural qualitative research on depression has been descriptive, documenting symptoms and explanatory models. There is a lack of qualitative research testing theoretical models of depression. The interpersonal model conceptualises grief, interpersonal disputes, role transitions and social isolation as the context in which depression develops and is the basis of interpersonal therapy (IPT), which is increasingly used in cross-cultural settings to treat depression. We aimed to qualitatively evaluate to what extent the interpersonal model can explain adolescent depression in Nepal. Data were collected between December 2018 and April 2019 and comprised transcripts from 126 participants: 25 semi-structured interviews with depressed adolescents aged 13–18; four focus group discussions with adolescents (N = 38), four with parents/caregivers (N = 39), and two with teachers (N = 17); and seven semi-structured interviews with health and non-governmental organisation workers. We coded data using an analytical framework comprising deductive codes representing key concepts from the interpersonal model of depression and IPT, including principles, techniques and strategies. Participants mainly related depression to interpersonal problem areas of grief, dispute, role transition and social isolation. Interpersonal disputes were common, and for many adolescents this involved parental physical and emotional abuse. Although role transitions were common few adolescents grieved loss of the prior role. Distress related to social isolation was evident despite close physical proximity and extensive social interaction with family and community members. Adolescents described coping strategies that were similar to strategies central to IPT, e.g. identifying helpful and unhelpful relationships and generating options and ways of managing problems. In conclusion, interpersonal problems are relevant to this population and conceptualisations align with core principles of the interpersonal model of depression. The findings highlight the importance of addressing abuse and maltreatment in depression aetiology. They also inform future cultural adaptations of IPT in Nepal and beyond, including the opportunity to integrate local coping strategies.

## Highlights

• Testing psychological models cross-culturally is important for adapting therapies.
• Interpersonal problems trigger depression among adolescents in Nepal.
• Experiences of depression align with core principles of interpersonal therapy (IPT).
• Few adolescents use IPT strategies to address interpersonal problems.
• Models of depression must incorporate poverty, migration and abuse as risk factors.

## 1 Background

Depressive disorders have been identified in cultures across the world, yet the majority of cross-cultural work has been descriptive in nature, typically focusing on similarities and differences in the phenomenology of symptom components of depression idioms (Haroz et al., 2017). Some work has also explored cultural models of causation and help-seeking (Aggarwal et al., 2014; Weiss et al., 1995). However, to date there has been limited work evaluating theoretical models of depression in cross-cultural populations, and almost none in non-Western adolescent populations.

There are a range of theoretical models of depression including early loss developmental models (Brown, 1982), psychodynamic models (neurotic child development) (Freud, 1966), stress-response models (both coping and HPA models) (Checkley, 1996), anhedonia-based (e.g., ‘stuck-in-a-rut’ functional neuroimaging (Holtzheimer and Mayberg, 2011), and positive/negative valence RdoC models (Pizzagalli, 2014)), behavioural (Lewinsohn, 1974) and learned helplessness (Seligman, 1975), Beck's Cognitive Theory (Beck, 1987), Hopelessness (Abramson et al., 1989), and Response Styles Theory (Nolen-Hoeksema, 1991).

Testing theoretical models cross-culturally is important to understand who is at risk of depression and how to develop and culturally adapt treatments informed by these models. Psychological treatments such as interpersonal therapy (IPT) and cognitive behaviour therapy (CBT) are concerned with helping patients to re-construct their interpretation of the world (Wampold, 2007). Existing cultural adaptations of these interventions have mainly focused on adaptation of peripheral aspects concerned with acceptability and feasibility of the intervention (e.g. provider, setting, group size and composition) (Chowdhary et al., 2014). Relatively few have addressed core aspects. These are the *therapeutic ingredients* associated with symptom change, based on psychological theory (Chu and Leino, 2017). Evaluating the extent to which a theoretical model of depression aligns with local experiences of depression can help to inform the selection and adaptation of treatment components for different settings (Heim and Kohrt, 2019).

One theoretical model of depression that has been informative for the development and adaptation of interventions is the interpersonal model (Klerman et al., 1984). It uses a diathesis-stress model of psychopathology and incorporates relational theory and findings from research on stress and social support (Lipsitz, 2013). It draws on the ideas of psychiatrists, Adolf Meyer and Harry Stack Sullivan, working in the early and mid-twentieth century, and on early attachment theory and the importance of a secure and trusting infant-caregiver bond (Mufson et al., 2004a). Contrary to Freudian psychoanalysis and its focus on the “intrapsychic”, Meyer viewed mental illness as an individual's maladaptation to their social environment. Sullivan's theory of emotions posited that mental disorders result from, and are perpetuated by, ineffective interpersonal communication. He suggested that an individual's actions should be understood in terms of their past and current social context.

The interpersonal model identifies four problem areas that can both trigger and maintain depression*:* interpersonal disputes, role transitions, grief, and interpersonal deficits linked to loneliness and social isolation (Weissman et al., 2018). Problems arise due to external, developmental and interpersonal factors that increase interpersonal stress, reduce social support, and create emotional difficulties. The relationship between interpersonal problems and depressive symptoms is reciprocal: emotional difficulties act to precipitate and maintain depression and in turn depression worsens interpersonal problems.

The interpersonal model has shaped the development of IPT, a diagnosis-targeted, time-limited psychological treatment developed in the 1970s to treat depression among adults (Weissman et al., 2000). IPT defines depression as a treatable medical condition. It focuses on links between mood and life events, and addressing difficulties related to the four interpersonal problem areas. IPT has been adapted for treatment of disorders such as bipolar disorder, bulimia nervosa and posttraumatic stress disorder, and for use among adolescents, the elderly and people with HIV (Lipsitz, 2013). It has been implemented in diverse cultural contexts such as China (Gao et al., 2010), Uganda (Bolton et al., 2003, 2007; Mutamba et al., 2018), Ethiopia (Asrat et al., 2020) and Hispanic populations in the US (Markowitz et al., 2009). The recent popularity of IPT coincided with its inclusion in the World Health Organisation (WHO) mental health Gap Action Programme (mhGAP) guide for the management of depression, and with the publication of the WHO group IPT intervention manual for low-resource, community settings (World Health Organization, 2016; World Health Organization and Columbia University, 2016). A systematic review of psychological interventions in low- and middle-income countries (LMICs) showed that interpersonal treatment elements across a range of therapies have the strongest effect size (Singla et al., 2017). Several mechanisms of change have been postulated, including reduced interpersonal stress, increased social support, and improved interpersonal skills and emotional processing (Lipsitz, 2013; Toth et al., 2013).

A systematic review of psychotherapy studies for adolescent depression globally showed IPT to be comparably effective to CBT, with some advantages in long-term effect and lower attrition (Zhou et al., 2015). IPT has shown evidence for efficacy and effectiveness in studies of depression among adolescents in both high- and LMICs (Bolton et al., 2007; Mufson et al., 2004b). The success of IPT in LMICs raises the question of whether the interpersonal model of depression is consistent with the experience, expression, and trajectory of depression in non-Western cultural groups. As opposed to cognitive-behavioural theories of depression, which focus on individual cognitive distortions and maladaptive behavioural patterns, a focus on interpersonal relations may have more cross-cultural universality. The goal of our study was to employ qualitative methods to evaluate how adolescent depression could be conceptualised using the interpersonal model in a non-Western youth population in rural Nepal. The study was part of a larger research programme to adapt and test the feasibility of IPT in this setting (Rose-Clarke, 2020).

## 2 Methods

### 2.1 Study setting

The study was undertaken as part of a broader project to explore the feasibility of group-based IPT for adolescents with depression in Nepal, in partnership with the non-governmental organisation Transcultural Psychosocial Organisation (TPO) Nepal. Adolescents aged 10–19 account for 23% of the Nepali population (Ministry of Health, 2016). We focused on adolescents because depression is one of the leading causes of Disability Adjusted Life Years in this age group, and because adolescents in Nepal are at high risk of depression due to recent and historical trauma (two major earthquakes in 2015 and a 10-year civil war between 1996 and 2006) on a background of socio-economic deprivation.

The setting was Sindhupalchowk, a rural mountainous district with a population of around 288,000. Sindhupalchowk was severely affected by the earthquakes in 2015: 2071 people were killed and many lost their homes and livelihoods (Government of Nepal). A study at this time found that 40% of adolescents met criteria for depression (Silwal et al., 2018). The district population is stratified into various caste/ethnic groups. Tamang ethnic groups comprise the largest group (34%) (UN Women, 2016). Brahman, Chhetri and Newar groups are the most socioeconomically privileged groups and account for 10%, 18% and 11% of the population respectively, whilst Dalit groups (7%) are the least privileged (UN Women, 2016). The main religions are Hinduism (59%) and Buddhism (38%) (Central Bureau of Statistics, 2011). Agriculture is the main source of income, though remittances from labour migrants are increasingly important (Ministry of Labour and Employment, 2016). Literacy rates among females and males are 52% and 68% respectively. Net school enrolment is 95% at primary, 78% at lower secondary and 43% at secondary level (UN Women, 2016).

### 2.2 Nepali ethnopsychology

Kohrt and Harper (2008) outline a Nepali ethnopsychology which identifies five elements of the self: *man* (heart-mind), *dimaag* (brain-mind), *jiu* (physical body), *saato* (spirit) and *ijjat* (social status) (Kohrt and Harper, 2008). The heart-mind and brain-mind are vital mental health concepts (Kohrt et al., 2012). The heart-mind is the site of emotion and memory, used to refer to feelings and *tension* (an English term widely used to refer to stress and mild psychological distress) (Kohrt and Harper, 2008; Kohrt et al., 2007, 2016). Being too emotional and having too much activity in the heart-mind can lead to physical and psychological illness. The term *manko samasyaa* (heart-mind problem) can be used to communicate feelings of anxiety and sadness or depression (*udas-chinta*) without incurring social stigma. Research suggests that heart-mind is understood by adolescents across genders and caste/ethnic groups in Nepal (Karki et al., 2009). The brain-mind functions in parallel with the heart-mind and represents the logical decision-making mind, controlling thinking and behaviour. Someone who acts in a socially inappropriate way (e.g. against gender or caste norms) is deemed to have a brain-mind problem (Kohrt et al., 2012). The terms *paagal* or *baulaahaa* (crazy, mad or psychotic) are used to describe someone with an extreme brain-mind problem. Individuals who are paagal are stigmatised; they and their family members are often socio-economically marginalised (Kohrt and Harper, 2008). Kohrt et al. (2012) used adult case studies to explore how Nepali ethnopsychology could be applied to an interpersonal framework (Kohrt et al., 2012). They suggested that heart-mind problems and behavioural control through the brain-mind are widely understood in the context of interpersonal relationships.

### 2.3 Data collection

We explored interpersonal models of adolescent depression in Sindhupalchowk. Data were collected between December 2018 and April 2019 and comprised 42 transcripts: 25 semi-structured interviews (SSIs) with adolescents aged 13–18 with depression (mean duration 41 min; range 25–67 min); four focus group discussions (FGDs) with adolescents aged 11–19 (N = 38), four with parents/caregivers (N = 39), and two with teachers (79 min; 40–114 min) (N = 17); six SSIs with health workers (auxiliary health workers, health assistant, midwife, doctor) and one with a representative from a local non-governmental organisation (43 min; 17–65 min). The Project Coordinator (male, MA Sociology) and Senior Research Associate (female, MA Sociology) conducted the SSIs and FGDs with two Nepali research assistants (RAs). The Project Coordinator and Senior Research Associate are experienced Nepali qualitative mental health researchers and conducted comprehensive training for the RAs prior to data collection.

School-going adolescents were recruited through government secondary schools. Out of school adolescents were identified through community stakeholders. We identified adolescents for the SSIs using a vignette of adolescent depression developed by Nepali and international clinicians, incorporating both ICD-11 and locally relevant symptoms (Jordans et al., 2015). RAs read the vignette to adolescents and invited those who felt they had similar symptoms for screening with the Depression Self Rating Scale (DSRS) and the Functional Impairment Tool (Birleson, 1981; Kohrt et al., 2011). Both tools have been validated in Nepal and we used established cut-off scores of ≥14 for the DSRS and ≥4 for functional impairment to identify adolescents with depression. Among those who screened positive we purposively sampled a range of caste/ethnic groups, in-and out of school and married and unmarried adolescents, across the full age range. We interviewed one adolescent who did not undergo screening but was interviewed on the basis of a diagnosis of depression made by our psychosocial counsellor. For FGDs we purposively sampled teachers, parents and adolescents across genders, caste/ethnicity and age group. Among health workers and the NGO worker we sampled a range of expertise. RAs conducted SSIs and FGDs in Nepali in a private space in a school, health post, or other community location.

We developed and piloted topic guides for SSIs and FGDs. The first part of the topic guides involved open questions and probes to explore participants' concepts and experiences of heart-mind problems as a proxy for depression, including effects on feelings and behaviour, coping, and help-seeking. We asked generally about perceived triggers of heart-mind problems, then probed specifically for grief, disagreements, loneliness and shyness, and life changes. The second part of the topic guide included questions to understand participants’ perceptions of a group psychological intervention for adolescents with depression.

King's College London (RESCM-18/19–8427) and Nepal Health Research Council (637/2018) provided ethical approval for the research. We explained the study (goals, reasons for doing the research) and distributed information sheets. We sought informed consent for all research participants as well as consent from a parent or caregiver for participants aged 17 and younger.

### 2.4 Data analysis

SSIs and FGDs were audio-recorded, transcribed into Nepali and translated into English for analysis. The quality of the data was regularly checked by the site lead by reading the Nepali transcripts whilst listening to the audio files. A standardised Nepali glossary was used to translate mental health terms (Acharya et al., 2017). The Framework Method was used to manage and analyse the data (Gale et al., 2013). We developed an analytical framework of deductive codes to reduce and summarise the data. The framework codified key concepts from Klerman and Weissman's original interpersonal theory of depression, was developed through a review of the literature on interpersonal models of depression and refined and approved by an IPT master trainer (Table 1) (Klerman et al., 1984). Codes related to interpersonal problems of grief, dispute, role transition and social isolation conceptualised as depressive “triggers”; core principles of the interpersonal model (e.g. depression is a medical illness; depression is curable); and generalised techniques and specific strategies related to each of the interpersonal problems that are used in IPT to alleviate depression and improve interpersonal relations. Codes related to the same concept were grouped together. Codes and categories helped to abstract the data, moving from individual accounts to a more general impression of the data. The first and second authors used the analytical framework to code three transcripts in parallel to test and refine the framework and check consistency in application. We divided the remaining transcripts and independently applied the analytical framework to code the rest of the data. We charted the coded data into a matrix developed using Microsoft Excel, which helped us to identify patterns and connections between and within codes and categories and across participants. We wrote notes to document the analysis, explore codes and categories, help guard against selectivity in use of the data, and examine our own roles, possible biases, and potential influence on the research. The first author combined the matrices, identified patterns under each of the codes and returned to the transcripts as a whole to address any gaps in the findings related to context and process. Findings were checked for relevance and trustworthiness by an IPT master trainer, IPT trainers working in Sindhupalchowk, and researchers who conducted the SSIs and FGDs.

**Table 1:** Analytical framework incorporating key components of an IPT model of depression.

CODE	Original description by Klerman and Weissman
Interpersonal stressors or triggers	
Grief	A complicated bereavement reaction following the death of someone known to the person
Disputes	An overt or covert disagreement with someone in the person's life
Role transitions	An unsettling change or expectation of change influencing personal relationships (e.g. an illness, birth of a child, marriage)
Social isolation	Longstanding difficulties in building and sustaining relationships
**Principles**	
Depression is a medical illness	Depression is a medical illness, rather than the patient's fault or a personal defect.
Depression is curable	Depression is a treatable condition
Reciprocal relationship of disorder and interpersonal context	Interpersonal problems trigger depression and depression triggers and/or exacerbates interpersonal problems.
Importance of losses of attachment in the development of depression	The importance of a secure relationship with a caregiver for warmth and nurture, and as a working model for interpersonal relationships in later life
**Supports and techniques**	
Linking mood to event and event to mood	Demystifying depression by understanding how mood and events are linked
Identifying helpful and unhelpful relationships	Identifying which relationships in a patient's life provide support, and which ones contribute to their problems
Identifying interpersonal problems related to current depression	Identifying whether grief, disagreements, life changes, or loneliness contribute to the onset of a depression episode
Understanding how communication affects others and how others' communication affects the person	Understanding how the content (verbal and non-verbal) and manner of communication affects others
Giving the sick role	Reducing the burden of current expectations on a depressed patient
Generating options and ways of managing life problems	Finding options to address problems will help the person to feel less hopeless and helpless.
**Strategies linked to problem areas**	
***Grief strategies***	
Becoming stronger in order to carry grief	Finding options to address problems will help the patient to feel less hopeless and helpless.
Linking relationship and circumstances to the grieving process	Understanding how the nature of the patient's relationship with the deceased, and the circumstances surrounding death, can affect the grieving process
Scheduling time to mourn	Setting aside regular time to mourn in order to remain functional at other times
***Dispute strategies***	
Understanding both sides of the argument	Trying to understand both sides of a disagreement
Identifying the phase of the dispute	Analysing the phase of the dispute (whether both parties are still negotiating, or are stuck in the dispute, or whether one or both parties want to end the relationship)
***Role transition strategies***	
Mourning the loss of the old role	Identifying the old role and mourning what has been lost
Identifying pros and cons of the new role	Identifying positive and negative aspects of the new role
Learning to manage the new role	Developing skills and building the resources needed to manage the new role
***Social isolation strategies***	
Changing habits to promote social engagement	Changing habits to end social isolation by increasing engagement with other people

Quality of the data collection, analysis and reporting was done in accordance with the COREQ standards (Tong et al., 2007). Supplemental Table 1 includes the COREQ item checklist.

## 3 Findings

The total sample comprised 126 participants representing the full range of caste/ethnic groups. Sample characteristics are presented in Table 2, Table 3, Table 4. Adolescents included in and out of school, and married and unmarried adolescents, from older and younger age groups.

**Table 2:** Characteristics of adolescent participants with depression (N = 25).

Median age (IQR, range)	15 (14–16, 13–18)
**Median Depression Self-Rating Scale score** (IQR, range) (N = 24)	19 (16.5–20.25, 14–32)
**Median Functional Impairment score** (IQR, range**)** (N = 24)	11 (9.75–14, 4–27)
**Females (%)**	14 (56)
**Married (%)**	1 (4)
**School going (%)**	23 (92)
**Has children (%)**	1 (4)
**Caste/ethnic group**	
Brahman/Chhetri (%)	13 (52)
Janajati including Tamang (%)	7 (28)
Dalit (%)	5 (20)

IQR Interquartile range.

**Table 3:** Characteristics of adult semi-structured interview participants (N = 7).

	N (%)
**Female**	4 (57.1)
**Caste/ethnic group**	
Brahman/Chhetri	4 (57.1)
Janajati	2 (28.6)
Madhesi	1 (14.3)
**Position**	
Auxiliary health worker	3 (42.9)
Auxiliary nurse midwife	1 (14.3)
Doctor	1 (14.3)
Health assistant	1 (14.3)
NGO worker	1 (14.3)

NGO Non-governmental organisation.

**Table 4:** Characteristics of focus group discussion participants.

Participant group	No. Of participants	Gender (M/F/Mixed)	Age range	Caste/ethnic groups
Adolescents	10	M	13–14	Brahman, Chhettri, Janajati, Dalit
Adolescents	11	M	13–19	Brahman, Chhettri, Janajati, Dalit
Adolescents	8	F	11–14	Brahman, Chhettri, Janajati, Dalit
Adolescents	9	F	16–18	Brahman, Chhettri, Janajati, Dalit
Teachers	9	Mixed	26–55	Brahman, Chhettri, Janajati, Dalit
Teachers	8	Mixed	28–57	Brahman, Chhettri, Janajati, Dalit
Fathers	12	M	21–66	Brahman, Chhettri
Fathers	8	M	31–60	Brahman, Chhettri
Mothers	10	F	25–40	Brahman, Chhettri, Janajati, Dalit
Mothers	9	F	24–61	Brahman, Chhettri, Janajati, Dalit

### 3.1 Stressors and triggers

Participants used a variety of terms to describe symptoms of depression, including the English words *tension* and *depression*, *man ko samasya* (heart-mind problem), *chinta* (anxiety), *manasik chintan* (problem with mental thinking), *aalchi* (lazy), *pagal* (mad)*, rogi* (frail). We asked depressed adolescents why they were experiencing these symptoms; interpersonal explanations were the first attribution for 19 out of 25 adolescents. Adolescents, parents, teachers and health workers attributed depressive symptoms to the death of a family member, having a poor financial situation, disability, love, quarrels in the family or with girlfriends or boyfriends, being beaten by family members, and being teased for belonging to a low caste. In 22 interviews with depressed adolescents the coding process revealed the presence of stressors related to the four interpersonal problem areas – dispute, grief, role transition and social isolation. In three interviews data were insufficient to identify a specific stressor or trigger. In five interviews coding revealed that adolescents linked their symptoms to more than one problem area. For example, a 14-year-old male linked his problems to a dispute with teachers involving physical abuse that forced him to transfer to a different school. He was finding the transition to a new school difficult in terms of adjusting to a different quality of education and socialising with new classmates.

### 3.2 Disputes

Thirteen depressed adolescents linked their problem to a dispute. Of these, ten described a dispute with their parents. Adolescents attributed disputes to being given an unfair amount of household work, making mistakes with their work, and parents being overprotective and refusing to let them play away from home and spend time with their friends. Parents and teachers attributed disputes to parents putting too much pressure on their children to study, disapproving of their children having boyfriends or girlfriends from another caste/ethnic group, and refusing to buy their children clothes or the resources they needed for school.

Two adolescents described how disputes were exacerbated when their mother or father drank alcohol.

*My father only scolds and shouts. He comes home drunk and beats [my] mother and when I go to separate them he calls me things like “randi” [bitch/whore] and so on. I get angry thereafter and there will always be a quarrel.* Female, aged 14.

Disputes often involved physical abuse: adolescents described being beaten or hit with a piece of wood by their parents. Some adolescents stayed away from home temporarily or permanently to avoid the violence.

Adolescents also described having disputes with their siblings, friends, girlfriends and teachers. A 16-year-old female described being *pagal* (mad), having severe headaches, pain and suicidal ideation, which she attributed to a dispute with a friend. She first visited a *dhami-jhankri* (traditional healer) who told her that she was possessed by evil spirits. She tried worshipping to help her recover but when there was no relief her father took her to the local hospital, and later to a mental institute where she received medication and “got better”.

Two adolescents linked their heart-mind problem to disputes that did not involve them directly. A 14-year-old female said that seeing her father beat up her mother made her angry, lose interest in food and stop talking with people. A 15-year-old male, who lived with his mother, brother and sister in rented accommodation, said that he was “tensed” because their landlord accused his mother of not paying rent and threatened to throw them out.

### 3.3 Role transition

Twelve adolescents with depressive symptoms described problems linked to a role transition. Seven described having to assume more household and/or paid work because one or both of their parents were unwell, had migrated, moved out of the family home, or were unable to earn sufficient funds to meet the family's basic needs. Adolescents whose parents were unwell related their tension to concerns about their parents' survival and having to abandon their plans to study in order to earn money. They worried about how to pay for their parents' medical care, as well as clothes, food and school fees for their younger siblings. A 16-year-old female described how her father and mother were unwell and neither were able to work. She had taken on agricultural work to contribute to the family's income. She explained how she would have to give up her education in order to ensure her elder and younger brothers could continue theirs. Three adolescents described role transitions related to a physical disability or chronic health condition. They were struggling to come to terms with functional impairment that affected their friendships and study. For example, a female adolescent described the impact of losing the ability to walk as a young child:

*Previously our school was located up a hill and we had to climb […] steps [to get there]. […] I used to think that I couldn't attend classes. I didn't want to talk with my friends. I also used to ask myself, why should I study when I cannot achieve anything in the future? […] While returning home from school I used to be last and everyone reached earlier than me. […] They'd say, “You cannot walk. If we go with her we will not reach home until evening.” When they said such things to me I used to get hurt. […] I used to think, when will I be able to walk like them? I can never be like them.*

Parents and adolescents described early marriage as a role transition and as a potential cause of heart-mind problems among adolescents:

*If they get married at such a playful age of 15-19, before 20, not the age of caring or supporting the children. After the child is born, they have to provide food and clothes, educate them, and protect and support them. Due to not being the appropriate age for doing all these things they get more stress and hence they can commit suicide.* Father.

### 3.4 Social isolation

Two adolescents with depression linked their symptoms to social isolation. A 17-year-old male belonging to the Dalit caste group, described feeling “sad belonging to a low caste” and uncomfortable with adolescents from other castes. His sense of social isolation was exacerbated as a result of living away from his family in rented accommodation in order to attend school. A male adolescent was “tensed” when his friends excluded him because of his skin condition:

*I got wounds all over my body. Those wounds were itchy and whenever I scratched them I got bigger wounds. […] My friends used to behave in a different manner with me, they used to get away from me, thinking that my wounds may transmit to them. They didn't let me play, even when I wanted to play.*

Other adolescents described situations of isolating themselves, for example staying alone, being absent from school, and not sharing their problems with family or friends, however from the interviews it was unclear whether depression caused the isolation or vice-versa.

### 3.5 Grief

*I found a case [having heart-mind problems] who was a student. […] His mother had just committed suicide and due to his father's weak condition he was having a hard time studying. He used to manage money for his study by buying barley from the market and selling the alcohol that he made from it. After that I discussed with the other teachers and we gave him a scholarship […]. I told him that he should study and we would help him.* Teacher.

Adolescents, parents and health workers identified the death of a family member, especially a parent, as a cause of depression. They described how bereaved adolescents faced financial hardship and were forced to discontinue their education and take on paid work and caring responsibilities, suggesting an overlap of grief and role transition problem areas.

Only one of the adolescents interviewed, a 16-year-old female, described a personal experience of grief. Her brother died during the earthquake and she attributed the thoughts she had been having in her heart-mind to the fact that she was missing her brother. Teachers described loss of a parent as a cause of heart-mind problems among adolescents, and included the death, migration and separation of parents in their definition of “loss”. The parallels between death and migration were evident in one 15-year-old girl's account of how she missed her mother who had migrated abroad when she was seven. Observing mothers and daughters in the community made her miss her mother even more.

### 3.6 Principles

IPT frames depression as a curable medical condition and not the person's fault. We found substantial evidence in support of a medical model of depression among adolescents who related the cause of depressive symptoms to “our mind or disease” and suggested visiting the doctor for treatment. They described physical symptoms of depression such as headaches and pain, and how emotions could have pathophysiological effects, for example anger affecting blood pressure. A health worker suggested depressive symptoms could be inherited:

*What I found in two or three cases was [that the] mother of two daughters hanged herself due to “depression”, a daughter hung herself at the age of 12, and the other daughter hung herself after one year. I did a post-mortem of both. After seeing the sequence I found that the symptoms are hereditary. A person can have a hereditary suicidal tendency for which we can do nothing.*

In contrast, teachers and fathers described how some individuals perceived depression as a *chhopne rog* (illness category suggesting being grabbed or caught by a spirit) due to “witch or devil effect” that could be treated by dhami-jhankris.

Evidence that participants perceived depression as a curable condition was inconclusive. Teachers said that adolescents with depression were locally referred to as *yo bigriyo, khattam nai bhayo* or *padhai khattam nai bhayo* (meaning spoiled), as well as *yo ta ava sakkiyo, siddhiyo* (meaning finished), implying an irrevocable condition. However, they also felt that some underlying problems could be solved.

*If the problem occurred due to poverty we cannot give them employment. We cannot possibly handle their financial problems in their houses. However, if the problem occurred due to lack of education and awareness, if they are ready for counselling, then [help is possible].* Teacher.

Parents, teachers and adolescents perceived depression as preventable by avoiding circumstances that might lead to stress and “tension” such as falling in love, or as controllable through demonstrating strength of character.

*In adolescence everyone will have such symptoms [of depression] and we should not fear it because this is the change brought by time. The person should be steadfast in their belief and not listen to others. They should keep their morale otherwise if they follow others they might spoil.* Mother.

The notion of a reciprocal relationship between depression and interpersonal problems is central to IPT. As described above, adolescents, teachers, parents and health workers attributed depressive symptoms to grief, disputes, social isolation and role transitions. However, few described how depression exacerbated interpersonal conditions and when they did it was mainly related to the effects of symptoms on daily functioning.

*Before I studied well and I used to be ranked first, second and third [in my class]. [Now] I feel like I don't have strength to think. […] I don't feel like doing anything and I also don't do anything I used to do before. I don't cook food or perform work like I used to do before. I only sleep in my room, don't speak to anyone and don't do homework.* Female, aged 14.

A 14-year-old male linked his heart-mind problem to being beaten and verbally abused by his teacher. Despite enrolling at a new school, he explained how whenever he felt “tensed and overburdened” he wanted to give up his studies. A 15-year-old female described how her parents wished she was a boy and at times this made her feel like leaving her studies or committing suicide. At other times she felt like studying hard or earning in order to prove herself.

IPT is predicated on attachment theory. The relevance of attachment problems was evident in adolescents’ depression history.

*My life has been miserable since early childhood. My parents used to drink a lot. They never cared how our lives would be. […] My mother used to beat me up while drunk and used to accuse me of having relations with my father. My father didn't hurt me as much as my mother did.* Female, aged 18.

Two adolescents recounted severe physical and verbal abuse perpetrated by their parents throughout their childhood. An 18-year-old female described seeking sanctuary with her grandparents and a 16-year-old male stayed with his uncle and aunt, however the extent to which these alternative caregivers were able to compensate for the lack of parental attachment was unclear.

Teachers and parents suggested that the absence of parents could lead to depression. Mothers described a unique openness between a mother and child, owing to the fact that the “mother gives birth”, and highlighted the importance of this relationship in childhood. Adolescents described seeking support and reassurance from their mothers when under stress. A 16-year-old female described how her mother listened to and reassured her. She stated that her “life wouldn't matter” in the absence of her mother.

Mothers and teachers also described the importance of fathers.

*My husband brought a second wife [home] whilst my child was small. Before that he was very clever and used to be carried or taken wherever my husband used to go. After my husband's second marriage [my child] became very slow and concentrated […]. It seems like his mind has altered or something is wrong with his heart-mind.* Mother.

### 3.7 Techniques and strategies

**Fig. 1:**
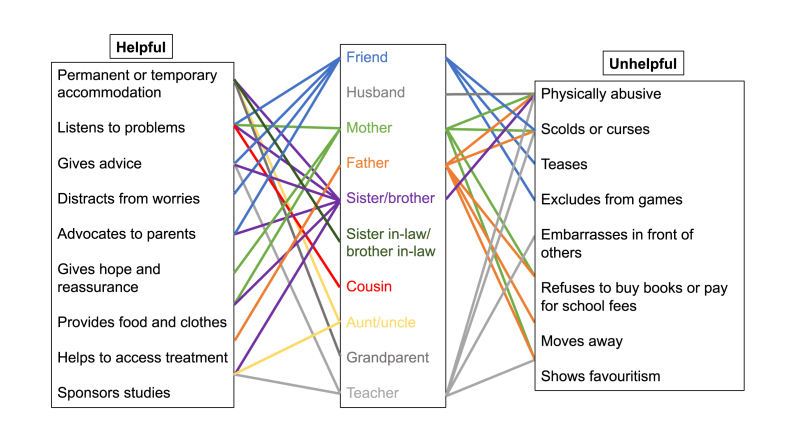
Helpful and unhelpful relationships described by adolescents with depression.

Table 5 summarises evidence from interviews with adolescents concerning their use of techniques and strategies relevant to IPT to alleviate depressive symptoms. All but three adolescents linked their mood to an interpersonal event. Most identified a helpful or unhelpful relationship in the context of their depression (Fig. 1). Adolescents described how siblings listened to their problems, gave advice, advocated to their parents, provided food and clothes, and sponsored their studies. They also described unhelpful relationships with fathers, mothers and teachers who scolded, cursed and physically abused them.

**Table 5:** Interpersonal therapy techniques and strategies used by adolescents with depression.

	General techniques						Grief strategies			Dispute strategies		Role transition strategies			Social isolation strategies
Participant number	Linking mood to event and event to mood	Identifying helpful/unhelpful relationships	Identifying interpersonal problems related to current depression	Understanding how communication affects others and how others' communication affects the person	Giving the sick role	Generating options and ways of managing life problems	Becoming stronger in order to carry grief	Linking relationship and circumstances to the grieving process	Scheduling time to mourn	Understanding both sides of the argument	Identifying the phase of the dispute	Mourning the loss of the old role	Identifying pros and cons of the new role	Learning to manage the new role	Changing habits to promote social engagement
**1**		●													
**2**	●	●	●												
**3**	●	●	●			●									
**4**	●	●	●	●						●					
**5**	●	●	●	●		●									
**6**		●													
**7**	●	●	●	●		●						●			
**8**	●	●	●	●											
**9**	●	●	●	●						●					
**10**	●	●	●	●							●				
**11**	●	●	●			●									
**12**	●		●											●	
**13**	●		●			●								●	
**14**	●	●				●									
**15**	●	●	●							●					
**16**	●	●	●	●											
**17**	●	●	●	●		●				●					
**18**	●	●	●			●					●				
**19**	●	●	●	●		●				●					
**20**	●	●	●	●		●								●	
**21**	●	●	●	●		●									
**22**	●		●	●											
**23**	●	●	●	●											
**24**	●	●	●	●											
**25**		●	●			●

Adolescents provided examples of how their mood had been affected by communicating with someone, for example *dukhkha lagne* (feeling sad) and *rish uthne* (feeling angry) when they were scolded, and *man halungo hune* (feeling lighter) and *fresh hune* (feeling fresher) when someone listened to their problems.

Twelve adolescents described how they had generated options to manage their life problems. Some had identified ways to manage their mood in the short term, including listening to music when they were tensed, or calling their siblings when they felt sad or suicidal. Others identified long-term approaches. A 13-year-old male worked as a labourer to help reduce his family's debt and a 15-year-old male worked to pay for his own health care.

We found no evidence that adolescents were given the sick role on account of their depressive symptoms. Many adolescents described inflexible daily routines: parents depended on their children's earnings and relied on them to do the household work and care for younger siblings. Only one female participant who suffered with chronic breathing difficulties described how her sisters and mother took on her household responsibilities when she was not feeling well, however this was related to her respiratory rather than depressive symptoms.

We searched for evidence that adolescents were implementing IPT strategies for grief, disputes, role transitions and social isolation. Among the two who linked their depressive symptoms to isolation, neither implied that they were trying to change their habits to promote social engagement. A 15-year-old female whose brother was killed in the earthquake did not describe using any IPT grief strategies. Instead she tried to manage her grief by staying alone in a peaceful place and sharing her thoughts:

*I share with my friends and parents. I ask my mother, “Why did it happen to us?“. I also share with my friends in school about whatever goes on in my heart-mind. […] I feel happy when they listen to me. My mother also listens to me, convinces me, and that makes me very happy. She convinces me saying that whatever happened in the past should be left behind […].*

Among those who linked their depressive symptoms to a dispute, we found evidence that they had attempted to understand both sides of the argument. A 14-year-old male left school when he was severely beaten by his teacher but acknowledged that the teacher “might have had some problem with herself as well”.

Identifying the stage of the dispute - negotiating, stuck, or wanting to end the relationship - is an IPT strategy to guide conflict resolution, and adolescents described disputes at each of these stages. An 18-year-old female described being stuck in a dispute with her husband: although she had moved out of his house due to physical abuse, she did not want to divorce him whilst her son was “too small”.

Of the 12 adolescents who described role transitions, only one described mourning the loss of their old role. A female adolescent developed difficulty walking and her mother had migrated outside Nepal. The adolescent recalled how, before these events she played in school and visited the community. Afterwards, she found it difficult to keep up with her friends and felt overburdened by the household work she was expected to do in her mother's absence. None of the adolescents suggested they had weighed up the pros and cons of their new role, but three described how they had learned to manage it. For example, because both of her parents were ill, a 16-year-old female participant had assumed new financial and caring responsibilities which she managed by finding paid work and helping her parents to attend health facilities.

## 4 Discussion

To the best of our knowledge, this study is the first to evaluate how depression could be conceptualised using the interpersonal model in a non-Western population. We found evidence that depression among adolescents in Nepal maps onto all four interpersonal problem areas of grief, dispute, role transition and social isolation. There was alignment with core IPT principles, for example some participants conceptualised depression as a medical illness. This appears to be a relatively new understanding of depression in Nepal, emerging from younger generations and the medical profession. Whilst participants intimated that depression was preventable, it was unclear whether they thought depression could be cured. We found evidence that adolescents were using some techniques and strategies common to IPT to improve depression (e.g. identifying helpful and unhelpful relationships and generating options and ways of managing problems). We found less evidence for the relevance of others, especially IPT strategies to address grief, role transitions and social isolation.

The triangulation of data from SSIs and FGDs and diversity of participants in terms of caste/ethnicity, age, role, and depressive status are strengths of this study. However, the study is limited in its focus on one district in Nepal. Data collection was not designed to explicitly elicit interpersonal models of depression. This was advantageous because participants were not prompted or influenced in describing their experiences of depression, however it also gave rise to gaps in our data. Although we probed about interpersonal triggers of depression we did not ask specifically about the use of IPT techniques and strategies and the extent to which they were or were not successful.

Our exclusive focus on the interpersonal model of depression could be a limitation in light of research suggesting Nepali ethnopsychology may also be compatible with CBT and dialectical behaviour therapy (DBT) models (Kohrt et al., 2012). However, it is worth noting that interpersonal explanations were the first attributions for depression for the majority of adolescents. There was almost no mention of one's thought patterns as responsible for heart-mind problems. This is consistent with Nepali ethnopsychology in which emotions, experiences, and memories are generated in the heart-mind, then processed in the brain-mind where cognition occurs. Therefore, changing one's experiences and relationships to impact emotions and thinking may be more culturally consistent than a focus on changing thoughts as the first step in alleviating distress. Regarding DBT, the focus on interpersonal relationships in that therapy may be why it has cultural appeal (Ramaiya et al., 2017). Other theories of depression are related to social comparison, social capital, and cultural consonance (Dengah, 2014; Gibbons, 1999; Song, 2015). These models are built upon subjective comparisons between one's status and others, or they examine how one's model of success or identity compares with others in the group. In adolescence the social comparison model may be particularly relevant because it is a crucial time for identity formation (Erikson, 1968). We found that adolescents in Nepal made many comparisons to others and related these to their depressive symptoms. For example, an adolescent from the Dalit caste group compared himself to peers from higher castes; an adolescent with a disability wished she was able to walk like her friends. Understanding whether the social comparison model of depression is a better fit than the interpersonal model could be the focus of future research.

We found no evidence that adolescents with depression were given the sick role. In settings where mental disorders are highly stigmatised, individuals labelled as depressed may be socially excluded leading to worsening of their symptoms. Furthermore, adolescents who self-label as depressed may experience higher levels of self-stigma and depression (Moses, 2009). According to the idiom of distress hypothesis, in collectivist societies such as Nepal somatisation can be a way to indirectly communicate distress and obtain support whilst preserving interpersonal relationships (Keyes and Ryff, 2003). Idioms of distress could therefore be an alternative to the sick role in adaptations of IPT in Nepal.

Few adolescents reported using IPT strategies for grief, disputes, role transitions and social isolation. This could be because: we did not probe for these strategies and hence they were not reported; because adolescents were not trying to address their problems; or because adolescents were using strategies aligned with other psychological models. For example, one adolescent described managing her grief by sharing her thoughts and feelings with her mother in order to leave the past behind. This approach may fit with cognitive behavioural models where the patient creates and shares their trauma narrative with a parent and converts their relationship with the deceased from interaction to memory (Cohen et al., 2006). Some adolescents described managing their tension by being alone and peaceful. DBT, with its emphasis on distress tolerance and mindfulness, may therefore be relevant in this setting (Kohrt et al., 2012).

In Nepal migration is common and often involves separation of parents from their families for several years at a time. The mental health and developmental impacts of migration on left-behind children have been well documented, however long term effects on attachment are less clear (Fellmeth G & Rose-Clarke K et al., 2018). Migration was described by some participants in our study in terms of the “loss” of a parent. One adolescent with depression described feelings similar to grief when her mother migrated. In an adaptation of IPT for adult Hispanic immigrants in the US, migration was categorised as a role transition (Markowitz et al., 2009). Left-behind children may also experience a role transition involving changes in family structure due to the absence of a parent (Mufson et al., 2004a).

We only identified two adolescents who attributed their depressive symptoms to social isolation although it is possible that others experienced social isolation causing or owing to their depression. In a context like Nepal (pre-COVID-19) physical isolation is unlikely as adolescents often live in large extended families, however social and emotional isolation are major stressors. Disputes and behaviour within the family, interrupted education, and lack of opportunities to socialise outside the home could isolate adolescents from their peers at a time where peer to peer relationships play a crucial role in development (Blakemore, 2018). Furthermore, owing to discrimination on the basis of caste or ethnicity there are circumstances where families may be socially isolated within a community (Kiang et al., 2020).

Several adolescents described interpersonal problems that cut across different problem areas. Many attributed their depressive symptoms to poverty which could be categorised under multiple problem areas. For example poverty could manifest as a role transition where an individual experiences the change from financially secure to insecure, or as a dispute where an individual compares their financial situation to others’ (indirect dispute) or experiences discrimination related to their low financial status (direct dispute). Participants described poverty as the mediating factor through which the death of a parent can lead to depression. In a South African adaptation of IPT, stress related to not being able to provide basic necessities was perceived to be directly related to depression and poverty was framed as a separate problem area (Petersen et al., 2012). Poverty has alternatively been conceptualised as a risk factor for depression where the acute trigger is either a role transition or dispute (Verdeli et al., 2003). An alternative model of depression among adolescents in Nepal might therefore situate interpersonal problems within a broader socio-ecological framework in which macro-level risk factors (poverty, economic migration, attitudes about interpersonal violence) shape interpersonal relationships.

Our findings highlight the importance of moving beyond simply categorising idiom differences in symptoms and description, to exploring how different theoretical models of depression fit with local experiences. This will help to inform cultural adaptations of successful interventions that go beyond contextualisation to include *deeper* changes to core content, increasing the likelihood of fit (Heim et al., 2019). We identified several co-existing explanatory models of depression including conceptualisation aligned with a medical model among adolescents and health workers, and more spiritual models among their parents and teachers. Future adaptations of IPT must be relevant to these models and ensure families, teachers and adolescents have a shared understanding of the goals and methods of IPT to reduce barriers to treatment. We suggest emphasising psychoeducation to raise awareness that depression is curable, which could help to give hope and reduce potential stigma towards those participating in IPT. We also recommend ethnographic research to better understand the social context in which some IPT strategies and techniques are used to reduce depression, whilst others are not. Group-based rather than individual IPT, where group members suggest approaches to address interpersonal problems, will help to ensure therapy is locally relevant and acceptable. Disputes interlinked with physical violence and child maltreatment were common. Adaptations of IPT must therefore make safety management an integral part of therapy. Narratives of poverty, and parental alcohol abuse and mental ill health were also common and highlight the need to integrate IPT into a broader package of care that links with child protection, educational support and social security schemes, and ensures parallel capacity building in adult mental health care.

In conclusion, we describe the first cross-cultural interpersonal model of depression among adolescents in Nepal. We demonstrate the relevance of interpersonal problems and provide evidence for alignment with core IPT principles. Our findings inform future cultural adaptations of IPT in this setting and beyond, including the need to integrate psychoeducation and local approaches to managing grief, disputes, role transitions and social isolation, and to link with health and social services. Our findings also highlight the importance of moving beyond categorisation and description of idioms of distress to explore how different theoretical models of depression fit with local experiences.

### 4.1 Data sharing

Due to the sensitive nature of the topic of this study and difficulties fully anonymising the transcripts data are not suitable for sharing.

## Role of the funding source

The study was co-funded by the UK Medical Research Council, the 10.13039/501100000272National Institute for Health Research and the 10.13039/501100002992Department for International Development (study reference: MR/R020434/1**)**. The funders had no role in the collection, analysis and interpretation of data; in the writing of the articles; and in the decision to submit it for publication.

## Author contributions

BK and KR-C conceptualised the study. All authors were involved in developing the analytical framework. PBK and JM supervised and participated in data collection. KR-C and EH conducted the analysis. KR-C wrote the first draft of the paper. All authors reviewed the paper and approved the final version for submission.
